# A Density-Driven Method for the Placement of Biological Cells Over Two-Dimensional Manifolds

**DOI:** 10.3389/fninf.2018.00012

**Published:** 2018-03-20

**Authors:** Nicolas P. Rougier

**Affiliations:** ^1^UMR5293 Institut des Maladies Neurodégénératives (IMN), Bordeaux, France; ^2^UMR5800 Laboratoire Bordelais de Recherche en Informatique (LaBRI), Talence, France; ^3^Inria Bordeaux - Sud-Ouest Research Centre, Talence, France

**Keywords:** stippling, Voronoi, spatial distribution, density, cells, neurons, retina, cortex

## Abstract

We introduce a graphical method originating from the computer graphics domain that is used for the arbitrary placement of cells over a two-dimensional manifold. Using a bitmap image whose luminance provides cell density, this method guarantees a discrete distribution of the positions of the cells respecting the local density. This method scales to any number of cells, allows one to specify arbitrary enclosing shapes and provides a scalable and versatile alternative to the more classical assumption of a uniform spatial distribution. The method is illustrated on a discrete homogeneous neural field, on the distribution of cones and rods in the retina and on the neural density of a flattened piece of cortex.

## 1. Introduction

**Figure 1:**
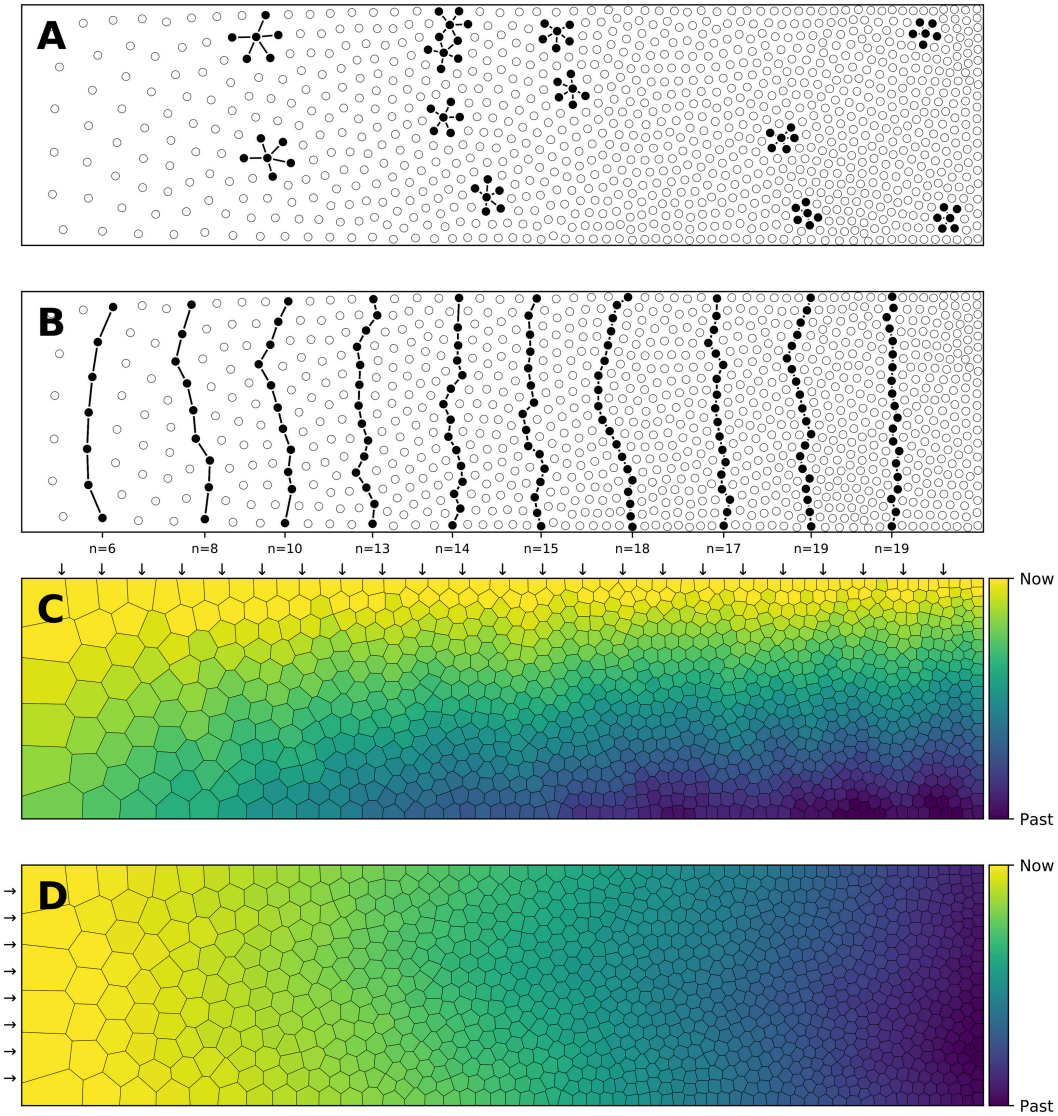
Influence of spatial distribution on signal propagation. **(A)** A k-nearest neighbors (*k* = 5) connectivity pattern shows mid-range connection lengths in low local density areas (left part) and short-range connection lengths in high density areas (right part). **(B)** Shortest path from top to bottom using a k-nearest neighbors connectivity pattern (*k* = 5). The lower the density, the shorter the path and the higher the density, the longer the path. On the far left, the shortest path from top to bottom is only 6 connections while this size triples on the far right to reach 19 connections. Said differently, the left part is the fast pathway while the right part is the slow pathway given some input data that would feed the architecture from the top. **(C)** Due to the asymmetry of the cell positions, a signal entering on the top side (indicated with small arrows) travels at different speeds and will consequently reach the bottom side at different times. This represents a spatialization of time. Color represents time. **(D)** Due to the asymmetry of the cell positions, a signal entering on the left side (indicated with small arrows) slows down while traveling before reaching the right side. This represents a compression of time and may serve as a short-term working memory. Color represents time.

**Figure 2:**
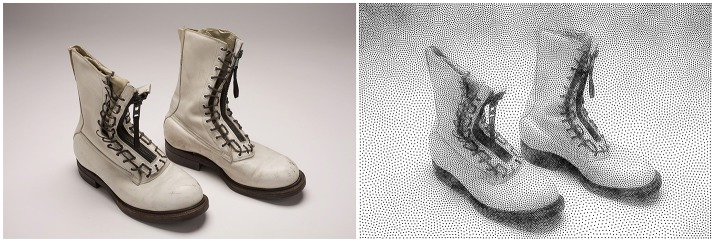
Stippling. According to Wikipedia^1^, *Stippling is the creation of a pattern simulating varying degrees of solidity or shading by using small dots. Such a pattern may occur in nature and these effects are frequently emulated by artists*. The pair of boots (left part) have been first converted into a gray-level image and processed into a stippling figure (right part) using the weighted Voronoi stippling technique by (Secord, 2002) and replicated in (Rougier, 2017). Image from (Rougier, 2017) (CC-BY license).

The spatial localization of neurons in the brain plays a critical role since their connectivity patterns may depend on their type and their position relatively to nearby neurons and areas (Ivenshitz and Segal, 2010). In the cortex, the probability of a connection existing between any two given areas declines sharply with distance (Markov et al., 2013), following an exponential decay with distance according to (Ercsey-Ravasz et al., 2013). For more local connections, such as interneurons, they generally have localized axonal arbors and interact mostly with close neighbors, depending on the distance (Jiang et al., 2015) from which a Gaussian probability of connection as a function of lateral distance can be derived (Potjans and Diesmann, 2012). Interestingly enough, whereas the neuroscience literature provides many data about the spatial distribution of neurons in different areas and species [e.g., Pasternak and Woolsey, 1975 about the spatial distribution of neurons in the mouse barrel cortex (McCormick et al., 2000) about the neuron spatial distribution and morphology in the human cortex (Blazquez-Llorca et al., 2014) about the spatial distribution of neurons innervated by chandelier cells], the computational literature exploiting such data is rather scarce and the spatial localization is hardly taken into account in most neural network models (be it computational, cognitive or machine learning models). One reason may be the inherent difficulty in describing the precise topography of a population such that most of the time, only the overall topology is described in terms of layers, structures or groups with their associated connectivity patterns (random, one to one, one to all, receptive fields, etc.). One can also argue that such precise localization is not necessary because for some models, it is not relevant (machine learning) while for some others, it may be subsumed into the notion of cell assemblies (Hebb, 1949) that represent the spatiotemporal structure of a group of neurons wired and acting together. Considering cell assemblies as the basic computational unit, one can consider local interactions to be subsumed into such assemblies and consequently, the exact spatial position of the neurons is not relevant. However, if cell assemblies allow to greatly simplify models, they also bring implicit limitations of which some have been highlighted in (Nallapu et al., 2017), such as for example the impossibility of having ambiguous representations (if such representations are identified with a single cell assembly) or to have topographic projections between two different groups. To overcome such potential limitations, we think the spatial localization of neurons is an important criterion worth to be studied because it could induce original connectivity schemes from which new computational properties can be derived as illustrated in Figure 1. However, before studying the influence of the spatial localization of neurons, it is necessary to first design a method for the arbitrary placement of neurons. This article introduces a graphical and scalable method for the automatic placement of neurons (or any other type of cells actually) enforcing a user-provided density map. This graphical method is based on a stippling technique originating from the computer graphics domain for non-photorealistic rendering as illustrated in Figure 2.

## 2. Methods

**Figure 3:**
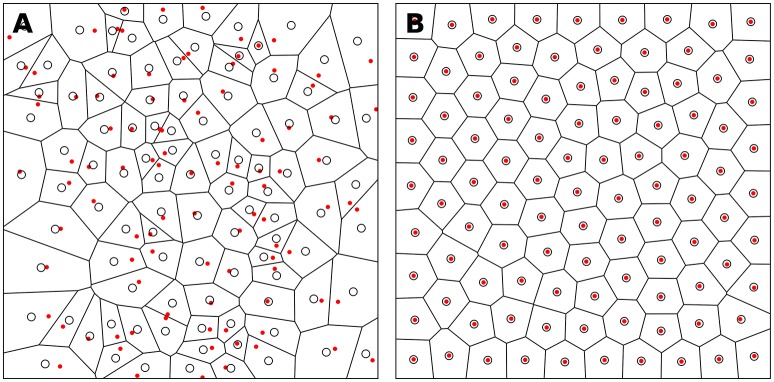
Centroidal Voronoi Tesselation. **(A)** Voronoi diagram of a uniform distribution (*n* = 100) where red dots represent the uniform distribution and white circles represent the centroids of each Voronoi cell. **(B)** Centroidal Voronoi diagram where the point distribution matches the centroid distribution which constitutes a blue noise distribution (i.e., *a distribution that is roughly uniformly random with no preferred inter-point directions or distances* according to the definition of Ebeida et al., 2014). This figure has been obtained from the initial distribution on the left after 50 iterations of the Lloyd relaxation algorithm.

Blue noise (Ulichney, 1987) is *an even, isotropic yet unstructured distribution of points* (Mehta et al., 2012) and has *minimal low frequency components and no concentrated spikes in the power spectrum energy* (Zhang et al., 2016). Said differently, blue noise (in the spatial domain) is a type of noise with intuitively good properties: points are evenly spread without visible structure (see Figure 3 for the comparison of a uniform distribution and a blue noise distribution). This kind of noise has been extensively studied in the computer graphics domain and image processing because it can be used for object distribution, sampling, printing, half-toning, etc. One specific type of spatial blue noise is the Poisson disc distribution that is a 2D uniform point distribution in which all points are separated from each other by a minimum radius (see right part of Figure 3). Several methods have been proposed for the generation of such noise, from the best in quality (dart throwing, Cook, 1986) to faster ones (rejection sampling, Bridson, 2007), see (Lagae and Dutré, 2008) for a review. An interesting variant of the Poisson disk distribution is an anisotropic distribution where local variations follow a given density function as illustrated in Figure 2 where the density function has been specified using the image gray levels. On the stippled image on the right, darker areas have a high concentration of dots (e.g., soles of the boots) while lighter areas such as the background display a sparse distribution of dots. There exist several techniques for computing such stippling density-driven patterns (optimal transport, Mehta et al., 2012, variational approach, Chen et al., 2012, least squares quantization, Lloyd, 1982, etc.) but the method proposed by (Secord, 2002) is probably the most straightforward and simple and has been replicated in (Rougier, 2017).

### 2.1. Centroidal voronoi tesselation

Considering a set of *n* points *P* = {_*P*_*i*_}*i*∈[1, *n*]_ on a finite domain *D* ∈ ℝ^2^, the Voronoi tesselation *V*(*P*) = {_*V*_*i*_}*i*∈[1, *n*]_ of *P* is defined as:

(1) $$\begin{array}{l} \forall i\in \left[1,n\right],V_{i}=\left\{x\in D|∥x-P_{i}∥\leq ∥x-P_{j}∥,\forall j\neq i\right\} \end{array}$$

Reciprocally, the (unique) Delaunay triangulation *T*(*P*) = {_*T*_*i*_}*i*∈[1, *n*]_ of *P* is the dual graph of the Voronoi diagram and defined such that no point in *P* is inside the circumcircle of any triangles in *T*(*P*). The centers of the circumcircles are equivalent to the Voronoi diagram, i.e., a partition of *D* into Voronoi cells. For each of the cell *V*_*i*_, we can compute its centroid *C*_*i*_ which is the center of mass of the cell. A Voronoi tesselation is said to be centroidal when we have ∀*i* ∈ [1, *n*], *C*_*i*_ = *P*_*i*_ (see Figure 3).

For an arbitrary set of points, there is no guarantee that the corresponding Voronoi tesselation is centroidal but different methods can be used to generate a centroidal tesselation from an arbitrary set of points. One of the most straightforward and iterative methods is the Lloyd relaxation scheme (Lloyd, 1982):

1. The Voronoi diagram of the *n* points is computed
2. The centroid of each of the *n* Voronoi cell is computed.
3. Each point is moved to the corresponding centroid of its Voronoi cell.
4. The method terminates if criterion is met (see below), else go to 1.

The algorithm finishes when the maximum distance between points and centroids is less than a given threshold as illustrated in Figure 3. It is to be noted that because of numerical imprecisions, there is no guarantee that an arbitrary small threshold can be reached.

### 2.2. Weighted centroidal voronoi tesselation

**Figure 4:**
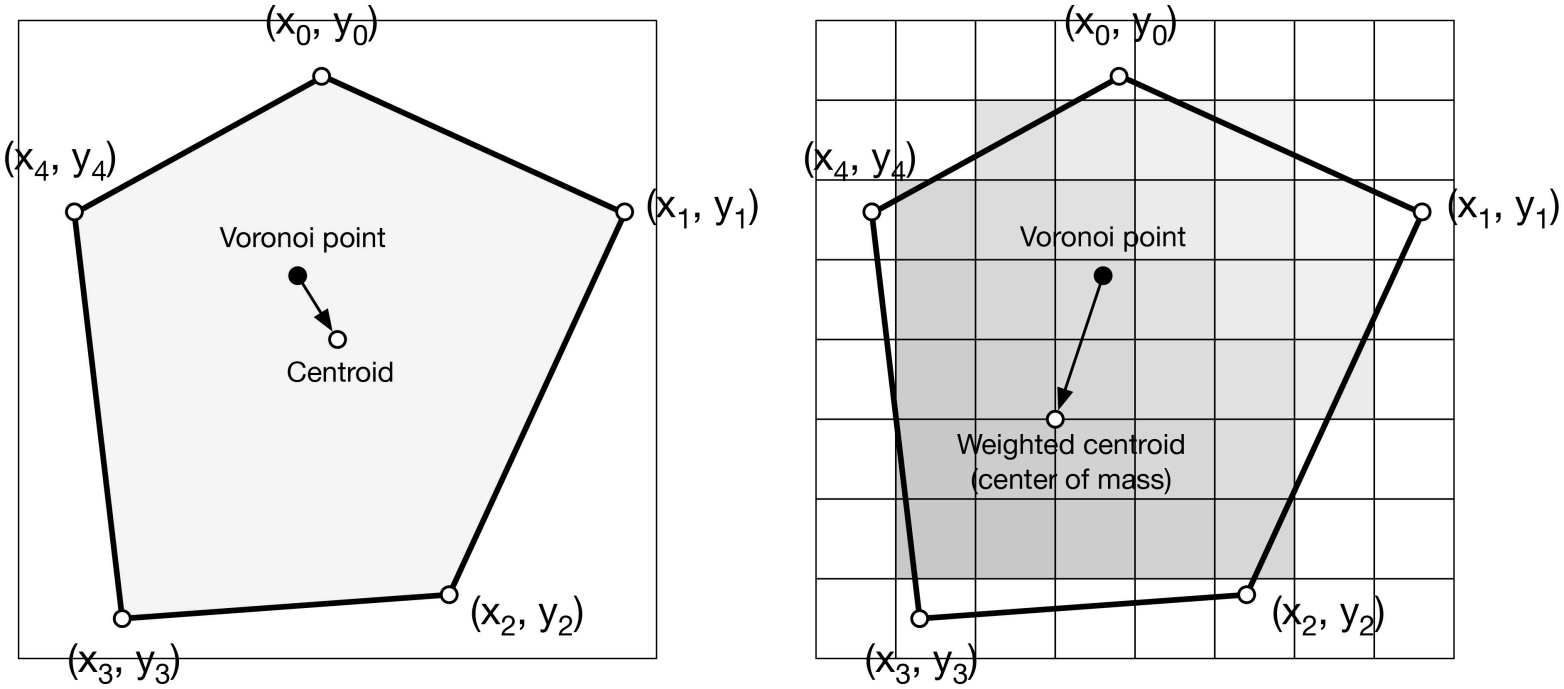
Weighted centroid. The weighted centroid of a Voronoi cell is the center of mass computed over the rasterized cell.

The weighted centroidal Voronoi tesselation, as illustrated in Figure 4, has been proposed in (Secord, 2002) and replicated in (Rougier, 2018). It is based on the Lloyd relaxation scheme with the notable difference that the centroids are now weighted according to the local density. This density information is provided using a bitmap image that represents the domain *D* ∈ ℝ^2^. Any of the RGB channels of the image can be used to provide the density information at a specific integer coordinate position. By arbitrary convention, we'll consider the darker color (e.g., black) to have the the higher density. The method is then as follows:

1. The density image is resized if necessary (no interpolation)
2. The Voronoi diagram of the *n* points is computed
3. Each Voronoi cell is rasterized as a set of pixels
4. The weighted centroid is computed over each of the rasterized cell
5. Each point is moved to the corresponding centroid of its Voronoi cell
6. The method terminates if criterion is met, else go to 2

A different criterion for the termination is to use a fixed number of iterations as we did for all the examples introduced in this article (*n* = 25).

**Figure 5:**
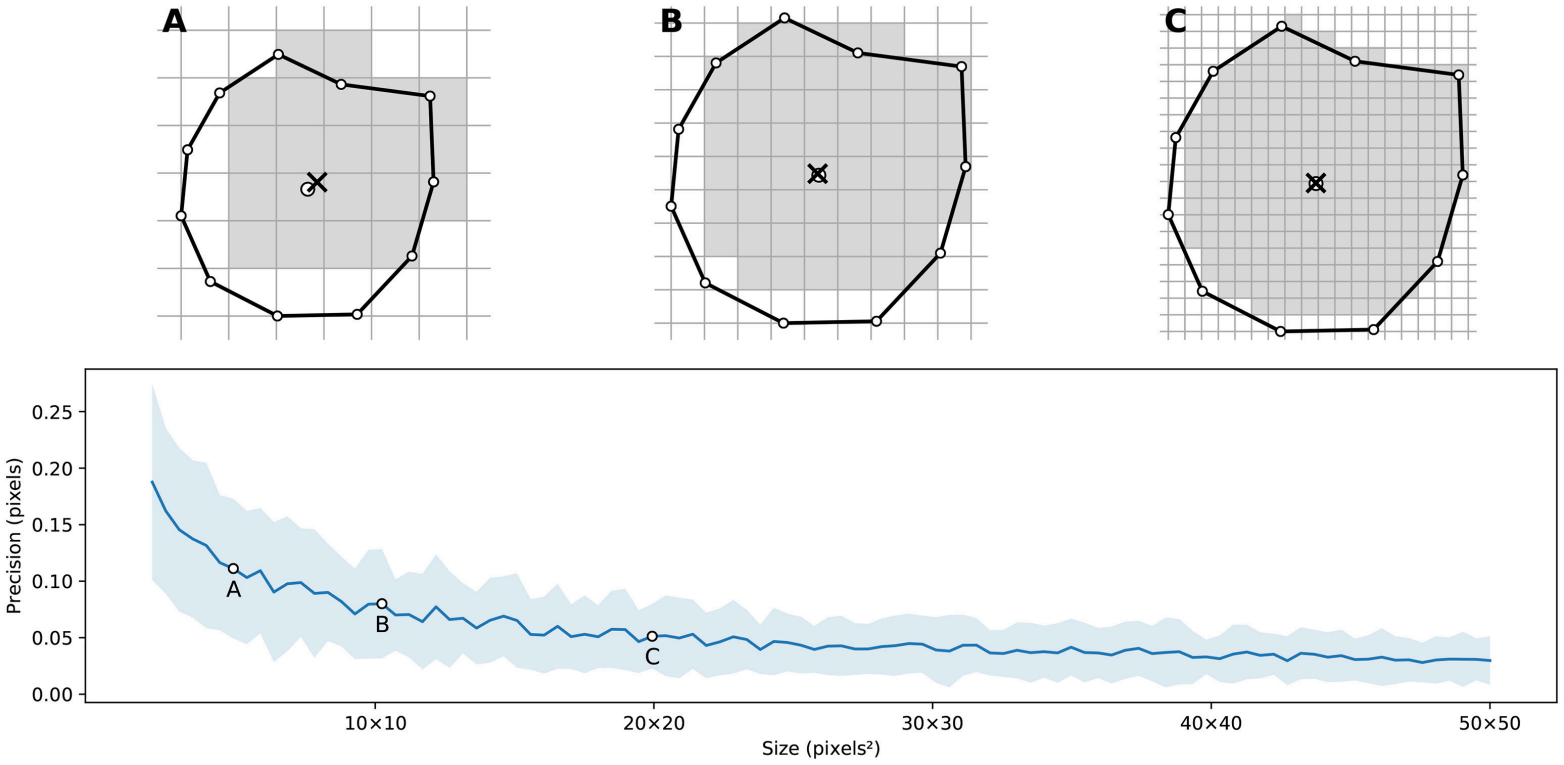
Rasterized centroid precision. The difference between the geometrical centroid (circle) and the centroid computed over the rasterized polygon (cross) is dependent on the size of the polygon. **(A)** 6 × 6 pixels rasterization. **(B)** 10 × 10 pixels rasterization. **(C)** 20 × 20 pixels rasterization.

Figure 4 illustrates the main difficulty in the method, that is, the rasterization of the cells and the computation of the weighted centroids. Since we use a bitmap image providing the density information and because the weighted centroids are computed over rasterized cells, it is quite obvious that the precision of the method is heavily dependent on the number of points and the size of the image. We estimated that a good precision can be reached if the mean number of pixels of a rasterized Voronoi cell is around 100 pixels (see Figure 5). For example, if we have initially 1,000 points to distribute and use a 100 × 100 input image, we would have only 10 pixels (100^*^100/1, 000) to compute the weighted centroid. Resizing first the image to 400 × 400 (without interpolation) makes this number to grow to 160 (400^*^400/1, 000). To obtain this 100 pixels estimation, we generated several polygons at different resolutions and compared the actual centroid (using its geometric definition) with the estimated centroid, considering a uniform density (whose center of mass is equal to the geometric centroid in such case).

**Figure 6:**
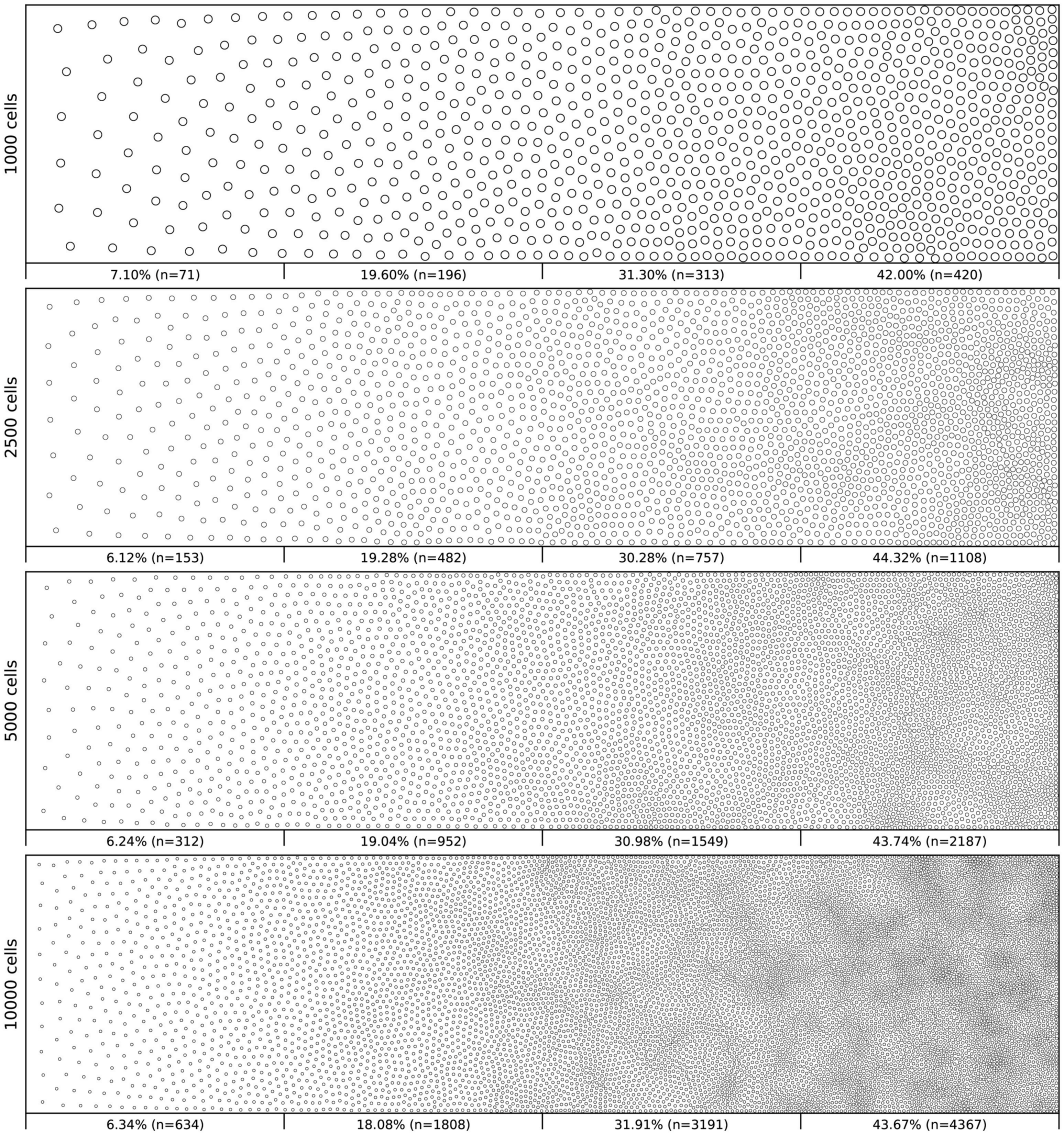
Non-uniform distribution (linear gradient). Different population distributions (size of 1,000, 2,500, 5,000 and 10,000 cells) using the same linear gradient as input have been computed. Each distribution has been split into four equal areas and the respective proportion and number of cells present in the area is indicated at the bottom of the area. The proportion of cells present in each area is approximately independent (±2.5%) of the overall number of cells.

Figure 6 shows the distribution of four populations with respective size 1,000, 2,500, 5,000 and 10,000 cells, using the same linear gradient as input. The local density is approximately independent of the total number of cells.

## 3. Results

We'll now illustrate the use of the proposed method on three different cases.

### 3.1. Case 1: retina cells

**Figure 7:**
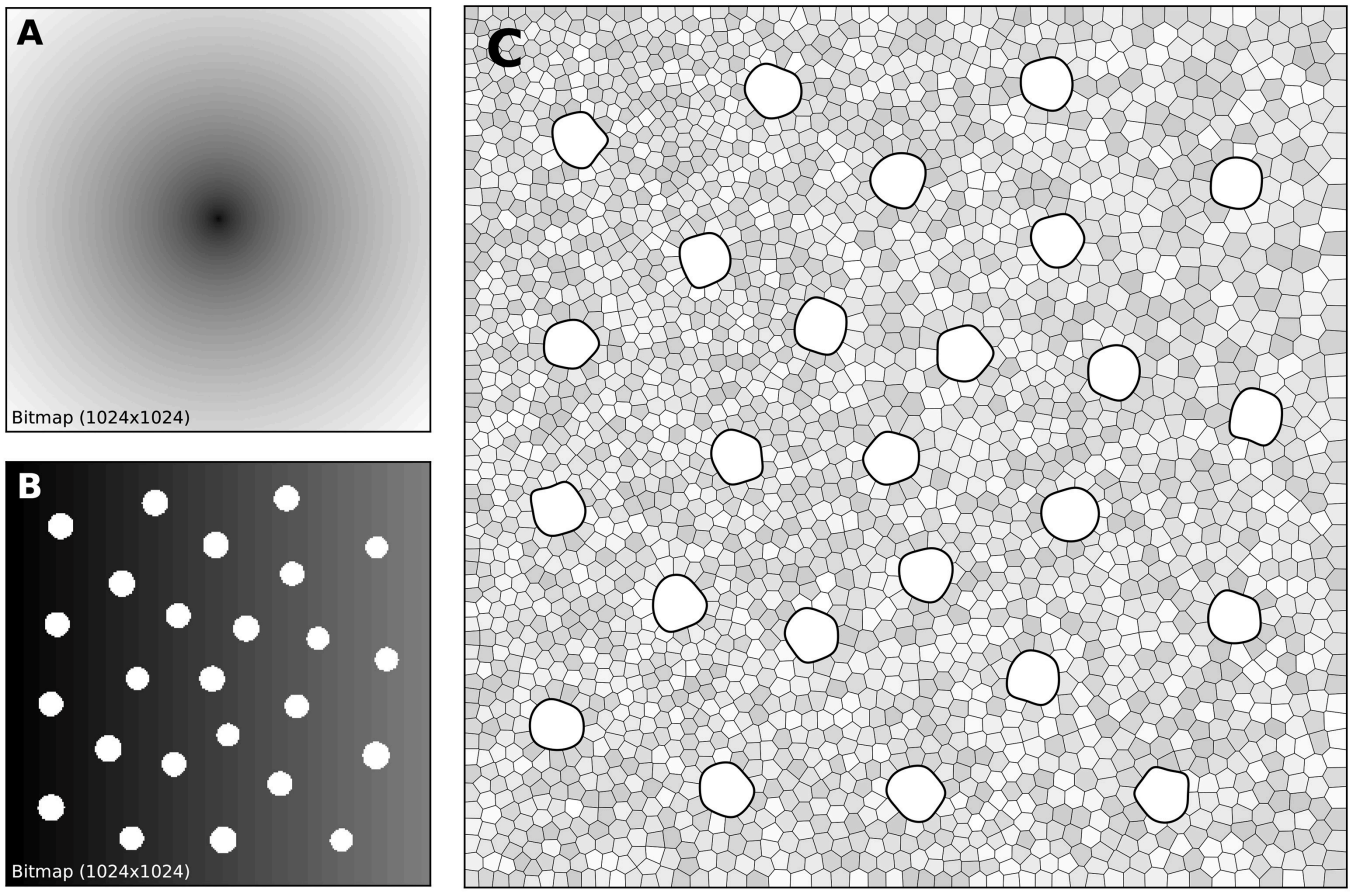
Cones and rods distribution. **(A)** The density map for the placement of cones (*n* = 25) is a circular and quadratic gradient with highest density in the center. **(B)** The density map for the placement of rods (*n* = 2,500) is built using the rods distribution. Starting from a linear density, *holes* with different sizes are created at the location of each cone and prevent rods from spreading over these areas during the stippling procedure. **(C)** Final distribution of cones and rods. Cones are represented as white blobs (splines) while rods are represented as Voronoi regions using random colors to better highlight the covered area.

The human retina counts two main types of photoreceptors, namely rods and cones (L-cones, M-cones and S-cones). They are distributed over the retinal surface in a non-uniform way, with a high concentration of cones (L-cones and M-cones) in the foveal region while the rods are to be found mostly in the peripheral region with a peak density at around 18–20° of foveal eccentricity. Furthermore, the respective size of those cells is different, rods being much smaller than cones. The distribution of rods and cones in the human retina has been extensively studied in the literature and is described precisely in a number of works (Curcio et al., 1990; Ahnelt and Kolb, 2000). Our goal here is not to fit the precise distribution of cones and rods but rather to give a generic procedure that can be eventually used to fit those figures, for a specific region of the retina or the whole retina. The main difficulty is the presence of two types of cells having different sizes. Even though there exist blue-noise sampling procedures taking different sizes into account (Zhang et al., 2016), we'll use instead the aforementioned method using a two stages procedure as illustrated in Figure 7.

A first radial density map is created for the placement of 25 cones and the stippling procedure is applied for 15 steps to get the final positions of the 25 cones. A linear rod density map is created where discs of varying (random) sizes of null density are created at the positions of the cones. These discs will prevent the rods from spreading over these areas. Finally, the stippling procedure is applied a second time over the newly built density map for 25 iterations. The final result can be seen in Figure 7C where rods are tightly packed on the left, loosely packed on the right and nicely circumvent the cones.

### 3.2. Case 2: neural field

Dynamic neural fields (DNF) describe the dynamics of a large population of neurons by taking the continuum limit in space, using coarse-grained properties of single neurons to describe the activity (Wilson and Cowan, 1972, 1973; Amari, 1977; Coombes et al., 2014). In this example, we consider a neural field with activity *u* that is governed by an equation of the type:

$$\tau \frac{\partial u\left(\text{x},t\right)}{\partial t}=-u\left(\text{x},t\right)+\int_{-\infty }^{+\infty }w\left(\text{x},\text{y}\right)f\left(u\left(\text{y},t\right)\right)d\text{y}+I\left(\text{x}\right)+h$$

The lateral connection kernel *w* is a difference of Gaussians (DoG) with short range excitation and long range inhibition that reads:

$$w\left(\text{x}\right)=I_{e}\exp^{\frac{-{\text{x}}^{2}}{\sigma_{e}}}-I_{i}\exp^{\frac{-{\text{x}}^{2}}{\sigma_{i}}}$$

The input *I*(**x**) is a scaled white noise that reads:

$$I\left(\text{x}\right)=I_{s}\;\times \;uniform\left(noise\right)$$

and the function *f* is a clamped linear function between 0 and 1 such that:

f(x)=max(min(x,1),0)

**Figure 8:**
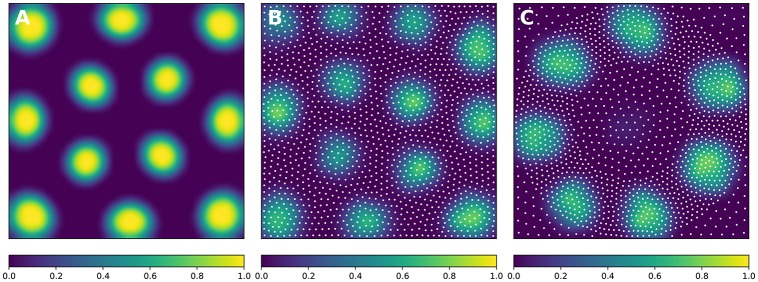
**Non-homogeneous discrete neural field**. Each plot has been smoothed using a bicubic filter. **(A)** Turing patterns resulting from a continuous and homogeneous neural field with constant and noisy input. **(B)** Turing patterns resulting from a discrete and homogeneous neural field with constant and noisy input. White dots indicate the position of the cells. Mean activity is computed from the histogram of neurons activity using 40 × 40 bins. **(C)** Localized Turing patterns resulting from a discrete and non-homogeneous neural field with constant and noisy input. White dots indicate the position of the neurons. Mean activity is computed from the histogram of neuron activity using 40 × 40 bins.

In order to solve the neural field equation, the spatial domain was discretized into a 40 × 40 grid, the temporal resolution was set to *dt* = 100*ms* and the simulation was run for *t* = 10 s. Relevant parameters are given in Table 1. In Figure 8A, one can see the characteristic Turing patterns that have formed within the field. The number and size of clusters depend on the lateral connection kernel. Figure 8B shows the discretized and homogeneous version of the DNF where each cell has been assigned a position on the field, the connection kernel function and the parameters being the same as in the continuous version. The result of the simulation shown in Figure 8B is the normalized histogram of cell activities using 40 × 40 regular bins. One can see the formation of the Turing patterns that are similar to the continuous version. In Figure 8C however, the positions of the cells have been changed (using the proposed stippling method) such that there is an annulus of higher density. This is the only difference with the previous model. While the output can still be considered to be Turing patterns, one can see clearly that the activity clusters are precisely localized onto the higher density regions. Said differently, the functional properties of the field have been modified by a mere change in the structure. This suggests that the homogeneous condition of neural fields (that is the standard hypothesis in most works because it facilitates mathematical analysis) is actually quite a strong limitation that constrains the functional properties of the field.

**Table 1:** Parameters for the neural fields.

**Parameter**	**Name**	**Value**
Grid size	n	40
Timestep	dt	100 ms
Duration	t	10 s
Time constant	τ	750 ms
Resting potential	h	0
Input scaling	*I*_*s*_	0.1
Noise level	N	0.1
Scaling factor	s	40^2^/*n*^2^
Sigma excitatory	σ_*e*_	0.05
Scale excitatory	*I*_*e*_	0.15 × *s*
Sigma inhibitory	σ_*i*_	0.085
Scale inhibitory	*I*_*i*_	0.05 × *s*

### 3.3. Case 3: cortical density

It has been shown in (Collins et al., 2010, Young et al., 2013, and Collins et al., 2016) that the neural density varies across and within cortical areas with an inverse relationship to the average neuron size: larger neurons take up more space and thus cannot be as densely packed as smaller neurons. (Collins et al., 2010) have studied the neural density in a cortical hemisphere of five primates and provided all the relevant data in the supplementary information. They dissected the flat hemisphere into a grid of 5 × 5mm piece and used an isotropic fractionator method to estimate the number of cells (neurons and non-neurons). To illustrate the method, we'll use the data from one of the two galagos that have been studied in order to produce a discrete distribution of sites enforcing the local measured density.

**Figure 9:**
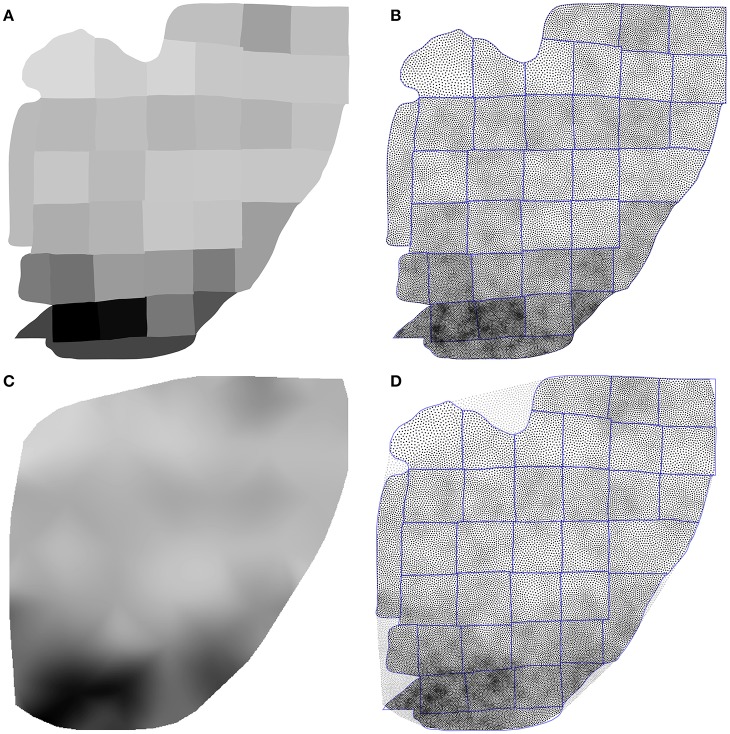
Flattened cortex. Data from (Collins et al., 2010), supplementary information. **(A)** Each cortex piece was assigned a gray level corresponding to the normalized density (density 1.0 being assigned to the most densely populated area). The result was saved as a 1, 000 × 1, 000 bitmap file (PNG). **(B)** Result of the stippling procedure for 25,000 sites and 25 iterations over the image generated in A. The mean difference for normalized density with actual data is 2.3% (±2.0%). Borders of the individual patches are drawn over the distribution (it is not an artifact). **(C)** Continuous cubic interpolation of the normalized density over the convex hull of A, using the centroid of each patch for computing the interpolation. The result was saved as a 1, 000 × 1, 000 bitmap file (PNG). **(D)** Result of the stippling procedure for 25,000 sites and 25 iterations over the image generated in C. The mean difference for normalized density with actual data is 2.9% (±2.8%). Outside sites (gray dots) are excluded and borders of the individual patches are drawn over the figure (it is not an artifact).

Using the Inkscape software^2^, we opened the supplementary information PDF file from (Collins et al., 2010) and isolated the top of the Figure S3 (galago 07-104). We renamed each individual patch according to the patch number indicated in the figure and saved the result as a SVG file. We took the first datasheet (galago 07-104) of the S1 dataset (Excel format) and converted it to a CSV format. Using the matplotlib library (Hunter, 2007), we produced a bitmap file (size 1000 × 1000 pixels see Figure 9A) where each cortical patch was drawn using a gray level that corresponds to its normalized density, a density of 1.0 (black color) corresponding to the most densely populated area (area 2).

Using the Shapely library (Gillies et al., 2007), we computed the convex hull of the whole set of the 36 cortical patches as well as the centroid for each individual patch. The boundary of the convex hull was resampled such as to have 50 equidistant points along the outline. The density information for these points was computed using the density of the nearest centroid. A cubic two-dimensional interpolation was computed inside the convex hull using a Clough-Tocher differential scheme (Alfeld, 1984) and the result was saved as a bitmap file (size 1000 × 1000 pixels, see Figure 9C). We'll refer to this interpolation as the continuous case.

The two bitmap files were processed with the provided stippler script (Rougier, 2018) using the red channel for density information and run over 25 iterations using N = {1000, 5000, 10000, 25000, 50000} sites. The result, for a single run, is a file with the 2-D coordinates of the *N* sites, the case for *N* = 25000 being shown on Figures 5C,D. From these coordinates, we computed the density for each of the original cortical patches by computing the patch area size and the number of sites inside. Results are indicated in Table 2. Unsurprisingly, the accuracy of the distribution grows with the number of sites (with one exception in the continuous case). For *N* = 50,000 sites, the difference between the actual density and the distribution is within a margin of 5%. In the continuous case however, it does not seem reasonable to expect a much higher accuracy than in the discrete (patch) case because the bitmap 1, 000 × 1000 has been interpolated using only 36 sites (patch centroids).

**Table 2:** Mean difference between the actual (normalized) density and the mean neural density using a patch bitmap (Figure 9A) and a continuous cubic interpolated bitmap (Figure 9C) for a various number of sites.

	**Patch**	**Continuous**	**Computation time**
*N* = 1,000	5.4% (±4.3%)	6.9% (±4.4%)	42s
*N* = 5,000	2.8% (±2.9%)	3.6% (±2.3%)	1m52
*N* = 10,000	2.8% (±1.8%)	4.0% (±3.0%)	2m55
*N* = 25,000	2.3% (±2.0%)	2.9% (±2.8%)	5m35
*N* = 50,000	0.8% (±0.6%)	2.6% (±2.0%)	8m44

*Computation times are only indicative and have been measured on a MacBook Pro with an Intel Core i7*.

## 4. Discussion

We've introduced a graphical method for the placement of biological cells over a two-dimensional manifold enforcing a density distribution that is provided using a bitmap image and the method has been illustrated on three simple use cases. For a more realistic placement (i.e., actual three dimensional structures), the method could be adapted but it is to be noted that several methods have been recently proposed. Parametric anatomical modeling (Pyka et al., 2014) allows one to model the anatomical layout of neurons as well as their projections while the work by (Schneider et al., 2014) allows one to go even further down by taking into account the dendritic morphology of neurons. However, due to its simplicity and beyond a strict biological plausibility, we think the proposed method might be interesting for a number of models, intermediate between symbolic models and realistic models. Our intuition is that such topography may be an important aspect that needs to be taken into account and studied in order for a model to benefit from structural functionality. For example, the Figure 1 shows the influence of the spatial distribution on the signal propagation when considering a simple nearest neighbors connectivity scheme. Even though such connectivity is unlikely to exist inside the brain, it might be nonetheless worth to be studied because it may provide structural functionality, that is, a function that directly derives from the topography.

## Notes

All figures were produced using the Python scientific stack, namely, SciPy (Jones et al., 2001), Matplotlib (Hunter, 2007), and NumPy (van der Walt et al., 2011). All sources are available on GitHub at github.com/rougier/density-driven (Rougier, 2018).

## Author contributions

NR designed and wrote the code, performed the experiments analysis and wrote the article.

### Conflict of interest statement

The author declares that the research was conducted in the absence of any commercial or financial relationships that could be construed as a potential conflict of interest.

## Footnotes

^1^Stippling Wikipedia entry at https://en.wikipedia.org/wiki/Stippling

^2^https://inkscape.org/en/
